# Effects of four cooking methods on sensory and taste quality of *Portunus trituberculatus*

**DOI:** 10.1002/fsn3.1398

**Published:** 2020-01-14

**Authors:** Shanshan Shi, Xichang Wang, Xugan Wu, Wenzheng Shi

**Affiliations:** ^1^ College of Food Science and Technology Shanghai Engineering Research Center of Aquatic Product Processing and Preservation Shanghai Ocean University Shanghai China; ^2^ College of Fisheries and Life Science Shanghai Ocean University Shanghai China

**Keywords:** cooking methods, electronic tongue, free amino acid, nucleotide, *Portunus trituberculatus*

## Abstract

The effect of heating medium (with water and steam) and initial temperature of heating medium (cold and boiling) on the taste quality and sensory properties of *Portunus trituberculatus* was investigated. Nonvolatile taste active components in the meat and gonad of crabs cooked under four different methods were detected and compared. Sensory evaluation and electronic tongue assessment were also conducted. Sensory evaluation and electronic tongue indicated that taste quality was different under four cooking methods. The results showed that steaming cooking preserved more water‐soluble substances as compared with boiling cooking, especially cooking began with boiling water. It is noteworthy that the umami intensity in gonad cooked from boiling water was stronger, either steaming or boiling. Therefore, this study can provide a theoretical basis and suggest the dynamic chemical changes of different organs during heating process need to be further studied.

## Graphical abstract

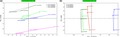

## 1 INTRODUCTION

The *Portunus trituberculatus* (Crustacea: Decapoda: Brachyura), also known as swimming crab, is the most widely fished species of sea crab, distributed in Southeast Asia, East Asia, Africa, the Americas, and Oceania, occupying an important position in China's coastal fishing economy, particularly in the Bohai and East China Sea.

Crab is considered as a prized dish in China, not only because of its delicious taste and uniquely pleasant aroma, but also for its rich nutritional qualities. Considerable researches have focused on the analysis of the chemical composition, nutrients, and flavor compounds of the edible parts of various fresh crab, including the green crab **(**Naczk, Williams, Brennan, Liyanapathirana, & Shahidi, 2004 **)**, Atlantic spider crab **(**Marques et al., 2010 **)**, Chinese mitten crab **(**Chen, Zhang, & Shrestha, 2007 **)**, and swimming crab **(**Jin, Xu, Qiu, & Fang, 2014 **)**. The edible quality of swimming crab is influenced by several intrinsic and extrinsic factors, and previous studies have demonstrated the effect of cultivation conditions, fattening periods, and storage conditions **(**Song, Wang, Wu, Wang, & Shi, 2018; Wu, Wang, Lou, Liu, & Cheng, 2014 **)**.

Taste quality plays a decisive role in consumers’ acceptance and consumption of food. Food technicians have long considered flavor enhancement as one of the most important means to improve the quality of food and in recent years. Today, many food products are heat processed, and the way in which we prepare food may have a significant impact on the quality of final product; much research has been done into the role of various cooking methods **(**Nieva‐Echevarrã­A, Goicoechea, Manzanos, & Guillã©N, 2018; Oz, Kaban, & Kaya, 2010 **)**. In China, consumers have four main ways for preparing fresh crabs; these are boiling started with cold water, boiling started with boiling water, steaming started with cold water, and steaming started with boiling water, respectively. But there is disagreement about which way the crab tastes better. Thus, it is necessary to analyze the taste properties and evaluate the impact of cooking conditions on the edible quality objectively combining with instruments. In regard to heating medium, traditional heat treatments include steaming and boiling; some people think steaming can preserve the flavor substances better, while others insist that boiled crab tasted better. Meanwhile, the initial temperature of heating medium was considered as another factor which induced different heating procedure; cooking began with boiling water means that heating crab sharply, while cooking began with cold water means heating crab gradually, and they induced different heat stress response. Previous studies have researched the gene expression of fish in response to heat stress **(**Heredia, 2008; Logan & Somero, 2011 **)**, but few studies investigated the heat shock response induced by different cooking methods. Ulrich reported that any organism experiencing temperature variation will also experience concomitant shifts in protein turnover rates and consequently protein synthesis and degradation pathways **(**Ulrich & Marsh, 2009 **);** thus, we hypothesized that initial temperature of heating medium may have a potential impact on the nitrogen compounds, consequently the taste quality.

The impact of cooking methods on the properties of the final product is interesting and worthy of investigation. So far, many studies have published on the effects of cooking methods on the nutritional quality and chemical safety of aquatic products **(**Ana Luísa et al., 2012; Gemma, Roser, Llobet, & Domingo, 2008 **);** they found the culinary treatments affected edible crabs’ chemical and elemental composition, but these studies are inadequate for sensory evaluation. To our knowledge, there is no information available describing the effect of the cooking methods on the taste quality and sensory properties of swimming crab. The aim of this study was to evaluate the possible effect of cooking methods for preparing swimming crab had on the edible quality. Combined with sensory evaluation, this study has more practical value for consumers and provides guidance for large‐scale production of crab in central kitchen.

## 2 MATERIALS AND METHODS

### 2.1 Sample preparation

A total of 120 swimming crabs (individually weighing 150–160 g) were picked and purchased from Luchaogang aquatic products market in Shanghai, China, and transported alive to the laboratory within 30 min in mid‐December 2018.

The crabs were soaked in plain water for approximately 30 min, before their shell surfaces were scrubbed clean with a brush and dried with kitchen paper. Drinking water (2 L) was added to four identical pots (Zhejiang Supor Co., Ltd). The swimming crabs were divided into four groups, according to their conditions for cooking, namely group BC (boiling started with cold water), group BB (boiling started with boiling water), group SC (steaming started with cold water), and group SB (steaming started with boiling water). The pots were then placed on the induction cooker and heated with 2000 W power, and the crabs cooked according to their designated conditions. The heating process was sustained for 15 min from boiling point. The claws, legs, and abdomen meat were picked by hand, and the crab meat was then minced and mixed and stored at −50℃ until analysis.

### 2.2 Electronic tongue

A lipid‐free extract was prepared in order to avoid damage to the potentiometric sensors. An accurately weighed sample (2.0 g ± 0.01) was homogenized in 25 ml distilled water for 1 min and sonicated for 5 min. The extract was stabilized for 30 min, then centrifuged (12,000 r/min, 15 min, 4°C), and the supernatant phase was collected and filtered. The steps were repeated in triplicate. The extracts were merged and diluted to 100 ml. Sensors were hydrated, preconditioned, calibrated, and diagnostics set prior to the analysis, according to manufacturers' instructions. The following parameters were used for auto‐sampler collection: Delay was 0 s, and acquisition time was 120 s. The sensors were rinsed in 80 ml distilled water for 10 s between each sample to avoid hysteresis effect.

### 2.3 Nucleotides

The nucleotides were extracted and detected according to a previously reported method **(**Ryder, 1985 **)** with slight modification. The specific steps were as follows: Sample (2.50 g ± 0.001) was homogenized in 10 ml (10%, v/v) cold perchloric acid (PCA), then subject to ultrasound for 5 min, centrifugated (4°C, 10,000 r/min, 15 min) to collect the supernatant, and the sediment was homogenized again in 5 ml PCA (5%, v/v). The procedure was repeated, and the upper layer collected and neutralized with 6 mol/L KOH. The sample solution was then filtered with 0.45 μm aqueous membrane. The entire process was operated at 0–4°C.

To achieve the nucleotide separations, HPLC conditions were set as follows: Inertsil ODS‐3 C18 (4.6 × 250 mm, 5 μm) liquid chromatography column, GL Sciences, column temperature: 30°C; flow rate: 1 ml/min; injection volume: 10 μl; UV detector wavelength: 245 nm. Mobile phase A was methanol, while phase B was phosphate buffer solution. The gradient elution procedure for HPLC was based on the method of Wang **(**Wang et al., 2015 **)**.

### 2.4 Free amino acids

Free amino acids were modified slightly according to the methods of Na **(**Na et al., 2013 **)**. Accurately weighed crab meat (0.5 g ± 0.01) and gonad (1.00 g ± 0.0001) were homogenized in 15 ml of 5% TCA and sonicated for 15 min, hydrolyzed in the refrigerator for 2 hr, then centrifuged (10,000 r/min, 4°C, 10 min) and filtered with 0.22 μm water phase filtration membrane.

Amino acids were detected by means of an automatic amino acid analyzer (L‐8800, Hitachi, Japan). The identity and quantity of the amino acids were assessed by comparison with the retention times and peak areas of the standard amino acids.

### 2.5 Equivalent umami concentration

The equivalent umami concentration (EUC, g MSG/100 g) is a quantitative index of the synergistic effect between the taste nucleotides (5'‐IMP, 5'‐GMP or 5'‐AMP) and monosodium L‐glutamate (MSG)‐taste amino acids (Asp or Glu), which expresses the umami intensity in terms of the concentration of MSG. According to Kawai **(**Kawai, Okiyama, & Ueda, 2002 **)**, it is expressed as the following equation as

$$\text{EUC}=\sum a_{i}b_{i}+1218\left(\sum a_{i}b_{i}\right)\left(\sum a_{j}b_{j}\right)$$

where, EUC value is the MSG equivalent (g MSG/100 g); a_i_ is the concentration (g/100 g) of Glu or Asp; *b_i_* is the relative umami concentration (RUC) of Glu or Asp relative to MSG (Glu:1, Asp:0.077); *a_j_* is the concentration (g/100 g) of taste nucleotides (5′‐IMP, 5′‐GMP, 5′‐AMP); *b_j_* is the RUC for each umami 5'‐nucleotide to 5'‐IMP (5′‐IMP: 1; 5′‐GMP: 2.3; 5′‐AMP: 0.18); and 1,218 is a synergistic constant based on the concentration of g/100 g used.

### 2.6 Sensory analysis

The sensory panel consisted of eight experienced students with no dietary bias and anaphylactic reaction (four female student and four male student, aged 22–25 years old). The carapaces and abdomens of the cooked swimming crabs were separated and placed in numbered plates for panelists to perform their sensory assessments. Using a quantitative descriptive analysis with slight modification **(**Stone, Sidel, Oliver, Woolsey, & Singleton, 2008 **; **Table 1), each panelist comprehensively scored four crabs cooked under different conditions according to their appearance, texture, odor, and taste in randomized order on a rating scale of 1–5 points, the larger the score, the stronger the characteristics. After assessing one sample, the panelists rinsed their mouths with pure water and crackers before tasting the next sample. Specific criteria for sensory evaluation are shown in Table 1.

**Table 1:** Sensory evaluation criteria for *Portunus trituberculatus*

Position	Sensory index	Score				
		1	2	3	4	5
Whole crab	Color	Poor(‐)	Poor	Medium	Good	Good(+)
	Texture	Poor(‐)	Poor	Medium	Good	Good(+)
	Odor	Poor(‐)	Poor	Medium	Good	Good(+)
	Taste	Poor(‐)	Poor	Medium	Good	Good(+)
Crab meat	Umami	Weak(‐)	Weak	General	Strong	Stronger(+)
	Sweetness	Weak(‐)	Weak	General	Strong	Stronger(+)
	Bitterness	Weak(‐)	Weak	General	Strong	Stronger(+)
Gonad	Umami	Weak(‐)	Weak	General	Strong	Stronger(+)
	Sweetness	Weak(‐)	Weak	General	Strong	Stronger(+)
	Bitterness	Weak(‐)	Weak	General	Strong	Stronger(+)

### 2.7 Statistical analysis

The results of this study were all subjected to an analysis of variance (LSD and Duncan) with the significant difference among means at the level of  = 0.05 using statistical package of SPSS version 21.0 (SPSS Inc., Chicago, U.S.A). Results were presented as means ± standard error. Taste intensity value (TAV) is the ratio between the determined concentration in the sample and its taste threshold value. Here, TAV used as the indicator to objectively assess the impact of compounds. The greater the value, the greater their contribution. When TAV > 1, the contribution of a substance to the overall taste is considered remarkable **(**Zheng, Tao, Gong, Gu, & Xu, 2015 **)**.

## 3 RESULTS AND DISCUSSION

### 3.1 Electronic tongue analysis

**Figure 1:**
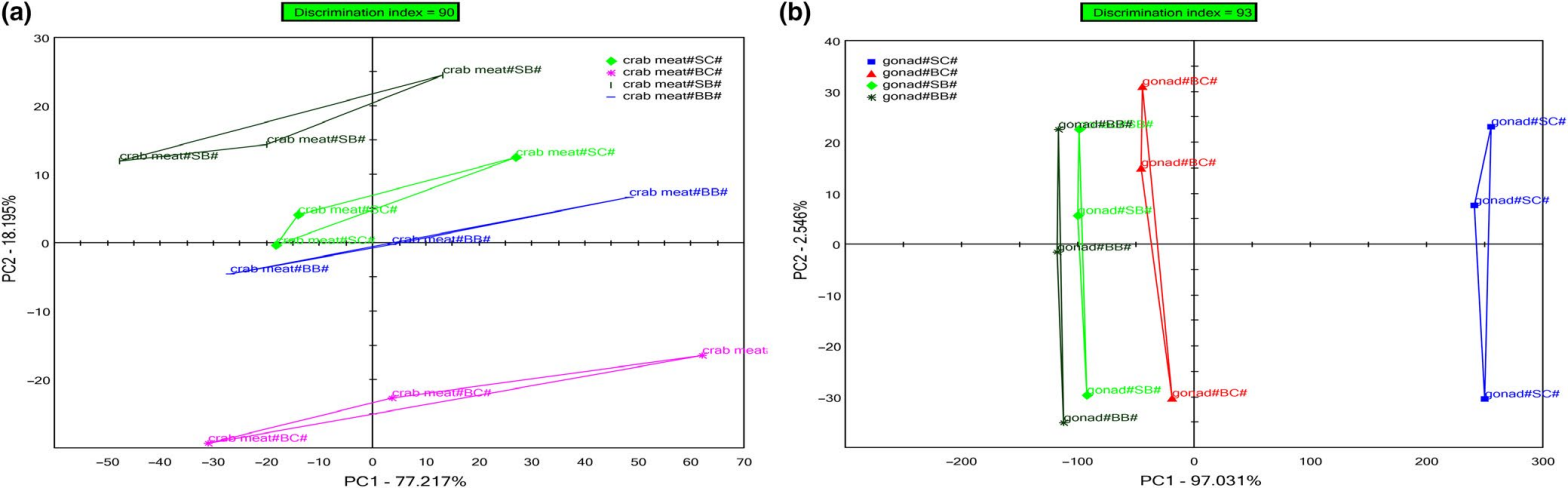
Perchloric acid chart for taste profiles of crab meat (a) and gonad (b) of *Portunus trituberculatus* cooked by different methods

Electronic tongue is a qualitative analysis technique by which samples are classified or identified, based on the composition of the taste sensor arrays. Unlike traditional methods, the electronic tongue does not obtain information about the nature of the compounds under consideration, but presents a digital pattern of the food material **(**Ghasemi‐Varnamkhasti, Mohtasebi, & Siadat, 2010 **)**. PC‐based real‐time control and a detection instrument are used to realize the automatic sample injection and data acquisition after initial set up, thus guaranteeing the repeatability and objectivity of the sensor testing compared with a sensory panel for routine food evaluation. Here, principal component analysis (PCA) was used (from the data generated by seven electrode sensors) to ascertain the classification of the taste profiles of the samples. Figure 1 shows the two‐dimensional scatter plots of the crab meat and gonad, in which the first two principal components together explain 95.41% and 99.58%, respectively, of the data variability, most notably 77.22% by PC1 (Figure 1a) and 97.03% by PC1 (Figure 1b). Consequently, the relationships observed between the samples in the scatter plots of variables can be considered representative of the relationships existing in the space of original data dimensions. Discrimination between the crab meat and the gonad cooked by different cooking methods was mainly determined by the X‐axis (PC1). The discrimination indexes (DI) were 90 (Figure 1a) and 93 (Figure 1b), with no observed overlap in the taste profiles, indicated differences between them. In Figure 1a, the crab meat of group BC is further away from the others, signifying that the taste characteristic of crab meat that boiling began with cold water was clearly distinguishable from the meat cooked by the other three methods. By contrast, in Figure 1b, the gonad of group BB is shown close to that of group SB, suggesting that gonad that started cooking with boiling water (both boiling and steaming) tasted similar.

### 3.2 Nucleotides analysis

Previous studies have reported the initial adenosine triphosphate (ATP) concentration in muscle is different from that in organs such as the ovaries or hepatopancreas **(**Hayashi, Asakawa, Yamaguchi, & Konosu, 1979 **)**. **Yang** reported, for example, that the concentration of nucleotides in the ovary of the female Chinese mitten crab was around twelve times greater than in its meat (Yang, 2006). Data presented in this study are consistent with above findings. The TAV of the three main flavor nucleotides is shown in Table 2. Quantitatively, AMP was found to be the nucleotide contributing most to the taste of crab meat, while TAV of guanosine monophosphate (GMP) and inosine monophosphate (IMP) was far <1, indicating that they made no significant contribution to the taste of the crab meat. Guanosine monophosphate and AMP contributed significantly to the taste of gonad, especially AMP, which was the major contributor. The TAV of AMP in the crab meat in group BC and group SC was about 1.27 times higher than that in group BB and group SB, while the contents of GMP, IMP, and AMP in the crab meat in group BC and group SC presented higher levels. Gorbatov's research suggested that different nucleotides have different levels of thermal stability **(**Gorbatov & Lyaskovskaya, 1980 **)**, resulting in different degree of thermal degradation and consequently, differences in their cooked content. Meinert similarly claimed that temperature would affect the degradation of nucleotides **(**Meinert et al., 2008 **)**. In this current study, it is presumed that high temperature inhibited the enzyme activity to degrade AMP, thus reducing the production of AMP in groups BB and SB. However, main nucleotides retained in the gonad did not exhibit the same phenomenon as the crab meat. Table 2 shows that cooking with boiling water from the start significantly improved the taste contributions of GMP and AMP to the gonad, which concurs with the conclusions of He's research **(**He, Cai, Wang, Yang, & Ding, 2017 **)**. This suggests that the temperature of cooking water is more important than the method of cooking (in this case, boiling or steaming) in determining the concentration of ATP breakdown products. In addition, it is speculated that the different stress mechanisms present in gonad and muscle may lead to different level of thermal degradation in ATP and its derivatives in swimming crab during food preparation. There may also be related to the Maillard reaction, a type of nonenzymatic browning; however, further analytical experiments are required to clarify the relationship between the preparation methods and changes in nucleotides in different organs.

**Table 2:** The TAVS of tasty nucleotides in crab meat and gonad of *Portunus trituberculatus* cooked by different methods

Main taste nucleotides	Threshold /(mg/100g)	TAV							
		Meat#BC	Meat#BB	Meat#SC	Meat#SB	Gonad#BC	Gonad#BB	Gonad#SC	Gonad#SB
GMP	12.5	0.22	0.14	0.13	0.15	3.73	3.99	3.36	5.33
IMP	25	0.28	0.16	0.2	0.23	0.49	0.45	0.35	0.69
AMP	50	1.73	1.38	1.74	1.36	5.99	7.04	5.74	9.17

### 3.3 Free amino acids analysis

The dominant free amino acids found in the crab meat were glycine (Gly), alanine (Ala), arginine (Arg), and proline (Pro). The high TAV of these amino acids indicated that they contribute significantly to the sweet taste of the cooked crab meat, which was consistent with the results of the sensory evaluation. Apart from the abovementioned amino acids, lysine (Lys), a bitter amino acid, was found to be abundant in the cooked gonad, which explains why it tasted bitter compared with the crab meat and is consistent with the results of the sensory evaluation. It can be seen from the TAV that glutamic acid (Glu), Ala, Lys, and Arg are the main contributors to the taste of gonad. Previous research has also identified the roles of certain amino acids, such as Gly and Pro, in the taste of cooked crab, with concentrations higher in marine than in freshwater species **(**Hayashi, Yamaguchi, & Konosu, 2010 **)**. It is noteworthy that the taste impact of Glu in this current study was strong due to its low threshold value (0.3) and synergistic effect with IMP, despite it being present in only a small quantity. Shahidi determined that Arg is found in many seafood products **(**Shahidi, Aishima, Abou‐Gharbia, Youssef, & Shehata, 1997 **)**. Although it has bitter characteristics, unlike those hydrophobic amino acids with branched chains that produce unpleasant bitterness, the presence of large amounts of Arg can provide food with a palatable flavor. The contents of other free amino acids are much lower than their corresponding threshold values, and the TAV values are far <1. However, it has been reported that some bitter amino acids can enhance the sweetness and taste of other amino acids when their concentration is below their threshold value, thereby improving the overall taste **(**Lioe, Apriyantono, Takara, Wada, & Yasuda, 2010 **)**.

The contents of total free amino acids (TFAA) in the crab meat and gonad cooked by different methods in the study are presented in Table 3. TFAA were divided into flavor amino acids (FAA) and essential amino acids (EAA) according to functional attributes. The FAA were further classified into umami (MSG‐like), sweet, bitter, and tasteless based on their taste properties as described in previous research literature **(**Beluhan & Ranogajec, 2011; Liu, Li, & Tangabcd, 2012 **)**. As shown in Table 3, the content of TFAA and FAA of steamed crab meat steamed was notably higher than that which was boiled. The highest concentration of TFAA was found in the crab meat of group SB, while the minimum concentration was obtained with the crabs whose boiling process started with cold water. The thermal denaturation and degradation of the protein were found to have significant impacts on the dynamic equilibrium of free amino acids. The thermal denaturation of the protein led to a decrease in free amino acids, along with the drip loss, whereas the increase of free amino acids was attributed to the thermal degradation of protein and polypeptides. Greater quantities of water‐soluble components were preserved in the steamed crab meat due to less drip loss, whereas the procedure of boiling is analogous to dilution. This was further validated by the turbidity of the soup, which was significantly clearer in the steaming pots than that it was in the boiling pots. Table 4 shows that the contents of TFAA in the gonad of group BB and group SB were higher than that of group BC and group SC. Unlike the crab meat, the cooked gonad's liquidity meant that is flowed and lost water, while the drip loss was reduced in the cooked gonad due to its lower water content. The gonad also firmed quickly under the hot water, and thus, the loss of water‐soluble components was relatively less. The present data gathered from the samples suggests, therefore, the boiling method is likely to release more nonvolatile components than the steaming method and that more nonvolatile components are retained at higher concentrations during steaming.

**Table 3:** The contents of free amino acids in crab meat of *Portunus trituberculatus* cooked by different methods

Amino acid species	Taste characteristics	Threshold/(mg/100g)	Concentration/(mg/100g)				TAV			
			Group BC	Group BB	Group SC	Group SB	Group BC	Group BB	Group SC	Group SB
Aspartic Asp^★^	Fresh/Sour (+)	1	11.74 ± 1.05^c^	12.68 ± 0.83^ab^	13.77 ± 1.82^ab^	15.89 ± 0.68^a^	0.12	0.13	0.14	0.16
Threonine Thr^▲^	Sweet (+)	2.6	74.46 ± 2.83^c^	84.4 ± 4.43^b^	66.57 ± 2.83^c^	134.7 ± 0.23^a^	0.29	0.32	0.26	0.52
Serine Ser^★^	Sweet (+)	1.5	3.41 ± 0.35^ab^	2.83 ± 0.59^c^	3.48 ± 0.25^ab^	4.18 ± 0.01^a^	0.02	0.02	0.02	0.03
Glutamic Glu^★^	Fresh/Sour (+)	0.3	31.5 ± 0.62^b^	27.56 ± 1.03^c^	31 ± 0.09^b^	34.75 ± 0.36^a^	1.05	0.92	1.03	1.16
Glycine Gly^★^	Sweet (+)	1.3	332.95 ± 11.81^ab^	297.85 ± 19.32^b^	384.54 ± 18.73^a^	360.97 ± 24.99^a^	2.56	2.29	2.96	2.78
Alanine Ala^★^	Sweet (+)	0.6	180.33 ± 1.22^bc^	151.62 ± 13.65^c^	198.29 ± 2.97^b^	234.48 ± 16.47^a^	3.01	2.53	3.3	3.91
Cysteine Cys	Bitter/Sweet/Sulfur (−)	‐	‐	‐	‐	‐	‐	‐	‐	‐
Valine Val^▲^	Sweet/Bitter (−)	0.4	29.8 ± 0.32^ab^	22.57 ± 0.68^b^	29.04 ± 0.74^ab^	36.14 ± 6.03^a^	0.74	0.56	0.73	0.9
Methionine Met^▲^	Bitter/Sweet/Sulfur (−)	0.3	26.58 ± 0.75^a^	21.84 ± 2.5^a^	26.65 ± 0.85^a^	28.03 ± 3.55^a^	0.89	0.73	0.89	0.93
Isoleucine Ile^▲^	Bitter(−)	0.9	14.66 ± 1.7^b^	8.4 ± 1.13^c^	15.48 ± 0.53^ab^	17.79 ± 0.12^a^	0.16	0.09	0.17	0.2
Leucine Leu^▲^	Bitter(−)	1.9	27.92 ± 1.08^b^	17.26 ± 1.92^c^	27.73 ± 1.35^b^	31.85 ± 0.85^a^	0.15	0.09	0.15	0.17
Tyrosine Tyr	Bitter(−)	‐	17.79 ± 3.63^a^	10.85 ± 2.25^b^	19.73 ± 0.87^a^	19.19 ± 0.25^a^	‐	‐	‐	‐
Phenylalanine Phe^▲^	Bitter(−)	0.9	22.46 ± 3.1^a^	11.69 ± 1.52^b^	24.51 ± 0.21^a^	23.21 ± 2.48^a^	0.25	0.13	0.27	0.26
Lysine Lys^▲^	Sweet/Bitter(−)	0.5	28.51 ± 0.53^a^	29.92 ± 7.36^a^	27.96 ± 1.32^a^	28.92 ± 3.35^a^	0.57	0.6	0.56	0.58
Histidine His	Bitter(−)	0.2	9.74 ± 0.62^b^	8.69 ± 0.18^b^	9.33 ± 0.05^b^	12.44 ± 0.43^a^	0.49	0.43	0.47	0.62
Arginine Arg	Sweet/Bitter (+)	0.5	555.64 ± 3.75^b^	570.31 ± 3.61^b^	561.2 ± 4.11^b^	646.96 ± 17.24^a^	11.11	11.41	11.22	12.94
Proline Pro^★^	Sweet/Bitter (+)	3	398.24 ± 1.76^a^	518.72 ± 21.09^a^	404.57 ± 31.14^a^	485.9 ± 100.29^a^	1.33	1.73	1.35	1.62
TFAA	‐	‐	1765.72 ± 35.12	1797.18 ± 82.07	1843.87 ± 67.85	2,115.39 ± 177.31	‐	‐	‐	‐
FAA	‐	‐	958.17 ± 16.8	1,011.26 ± 56.5	1,035.66 ± 55	1,136.17 ± 142.8	‐	‐	‐	‐
EAA	‐	‐	224.39 ± 10.32	196.07 ± 19.53	217.94 ± 7.82	300.63 ± 16.6	‐	‐	‐	‐
FAA/TFAA(%)	‐	‐	54.27	56.27	56.17	53.71	‐	‐	‐	‐
EAA/TFAA(%)	‐	‐	12.71	10.91	11.82	14.21	‐	‐	‐	‐

Note

All data were presented as means ± standard error. ★represents flavor amino acids; ▲represents essential amino acids; ‐represents no detected; TFAA means total free amino acids; FAA means flavor amino acids; EAA means essential amino acids.

Means of content with different letters within each row were significantly different (*p* < .05) regarding cooking methods effect.

**Table 4:** The content of free amino acids in gonad of *Portunus trituberculatus* cooked by different methods

Amino acid species	Taste characteristics	Threshold/(mg/100g)	Concentration/(mg/100g)				TAV			
			Group BC	Group BB	Group SC	Group SB	Group BC	Group BB	Group SC	Group SB
Aspartic Asp^★^	Flesh/Sour (+)	1	7.07 ± 0.13^c^	11.25 ± 0.45^b^	12.26 ± 0.01^b^	15.58 ± 1.99^a^	0.07	0.11	0.12	0.16
Threonine Thr^▲^	Sweet (+)	2.6	24.98 ± 0.31^d^	34.94 ± 0.37^b^	30.83 ± 0.84^c^	49.37 ± 0.5^a^	0.1	0.13	0.12	0.19
Serine Ser^★^	Sweet (+)	1.5	4.83 ± 0.04^d^	6.37 ± 0.48^b^	5.57 ± 0.01^c^	8.05 ± 0.21^a^	0.03	0.04	0.04	0.05
Glutamic Glu^★^	Flesh/Sour (+)	0.3	27.63 ± 0.24^b^	40.01 ± 0.15^a^	30.19 ± 0.16^b^	35.62 ± 3.28^a^	0.92	1.33	1.01	1.19
Glycine Gly^★^	Sweet (+)	1.3	64.35 ± 0.35^d^	106.14 ± 4.86^a^	90.67 ± 0.38^b^	75.63 ± 3.82^c^	0.5	0.82	0.7	0.58
Alanine Ala^★^	Sweet (+)	0.6	59.6 ± 0.43^c^	84.59 ± 7.98^a^	69.23 ± 0.44^bc^	77.41 ± 6.24^ab^	0.99	1.41	1.15	1.29
Cysteine Cys	Bitter/Sweet/Sulfur (−)	‐	‐	‐	‐	‐	‐	‐	‐	‐
Valine Val^▲^	Sweet/Bitter (−)	0.4	16.97 ± 0.12^c^	22.21 ± 1.04^a^	21.91 ± 0.04^a^	19.21 ± 0.09^b^	0.42	0.56	0.55	0.48
Methionine Met^▲^	Bitter/Sweet/Sulfur (−)	0.3	13.16 ± 0.01^b^	18.17 ± 1.47^a^	17.29 ± 0.3^a^	14.16 ± 0.13^b^	0.44	0.61	0.58	0.47
Isoleucine Ile^▲^	Bitter(−)	0.9	11.58 ± 0.09d	13.67 ± 0.34^b^	14.61 ± 0.09^a^	12.55 ± 0^c^	0.13	0.15	0.16	0.14
Leucine Leu^▲^	Bitter(−)	1.9	21.68 ± 0.05^c^	27.24 ± 0.72^b^	28.94 ± 0.03^a^	27.37 ± 0.84^b^	0.11	0.14	0.15	0.14
Tyrosine Tyr	Bitter(−)	‐	24.64 ± 0.03^b^	30.24 ± 2.81^a^	29.2 ± 0.22^a^	31.03 ± 0.86^a^				
Phenylalanine Phe^▲^	Bitter(−)	0.9	24.07 ± 0.55^b^	30.27 ± 2.57^a^	29.96 ± 0.51^a^	31.58 ± 0.27^a^	0.27	0.34	0.33	0.35
Lysine Lys^▲^	Sweet/Bitter(−)	0.5	81.22 ± 0.36^c^	133.56 ± 8.37^a^	98.56 ± 0.25^b^	108.5 ± 3.87^b^	1.62	2.67	1.97	2.17
Histidine His	Bitter(−)	0.2	11.37 ± 0.02^c^	17.48 ± 0^b^	16.16 ± 0.07^b^	19.15 ± 1.17^a^	0.57	0.87	0.81	0.96
Arginine Arg	Sweet/Bitter (+)	0.5	231.61 ± 3.2^c^	365.17 ± 0.06^a^	324.44 ± 1.82^b^	347.03 ± 20.06^ab^	4.63	7.3	6.49	6.94
Proline Pro^★^	Sweet/Bitter (+)	3	63.97 ± 0.7^c^	111.63 ± 10.27^a^	81.06 ± 0.07^b^	86.09 ± 3.46^b^	0.21	0.37	0.27	0.29
TFAA	‐	‐	688.73 ± 6.64	1,052.92 ± 41.93	900.88 ± 5.23	958.31 ± 46.8	‐	‐	‐	‐
FAA	‐	‐	227.45 ± 1.89	359.98 ± 24.19	288.98 ± 1.06	298.37 ± 19.01	‐	‐	‐	‐
EAA	‐	‐	193.66 ± 1.5	280.06 ± 14.88	242.09 ± 2.05	262.74 ± 5.7	‐	‐	‐	‐
FAA/TFAA(%)	‐	‐	33.03	34.19	32.08	31.14	‐	‐	‐	‐
EAA/TFAA(%)	‐	‐	28.12	26.6	26.87	27.42	‐	‐	‐	‐

Note

All data were presented as means ± standard error. ★represents flavor amino acids; ▲represents essential amino acids; ‐represents no detected; TFAA means total free amino acids; FAA means flavor amino acids; EAA means essential amino acids.

Means of content with different letters within each row were significantly different (*p* < .05) regarding cooking methods effect.

### 3.4 Equivalent umami concentration analysis

**Figure 2:**
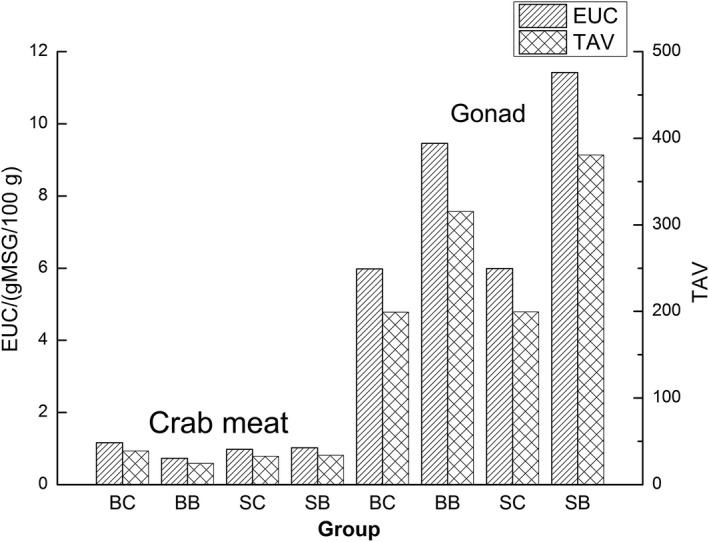
Equivalent umami concentration values of crab meat and gonad of *Portunus trituberculatus* cooked by different methods

As shown in Figure 2, the gonad was found to be tastier than the crab meat in this study, which concurs with the findings reported for raw Chinese mitten crab **(**Chen & Zhang, 2007 **)**. The higher EUC values of gonad cooked in groups BB and SB revealed that the umami intensity of the gonad cooked began with boiling water was higher than the other two groups, while the EUC values of cooked crab meat were almost equal (Figure 2). The results further show that the EUC value of gonad correlates with the initial temperature of heating medium but little effected by the heating medium.

### 3.5 Sensory analysis

As shown in Table 5, sensory results showed that the cooking mode had little impact on color, texture, odor, and taste scores (*p* > .05). The sensory results indicated there was no significant difference on the umami taste, sweetness, and bitterness of crab meat under different cooking methods (*p* > .05). By contrast, the taste characteristics of the gonad varied remarkably under the different cooking methods (*p* < .05). Cooking began with boiling water, the gonad presented intensified umami taste. The results of the sensory analysis were correlated with the EUC values and electronic tongue.

**Table 5:** Effects of different methods on the sensory evaluation of crab meat and gonad of *Portunus trituberculatus*

Group	Whole crab				Crab meat			Gonad		
	Color	Texture	Odor	Taste	Umami	Sweetness	Bitterness	Umami	Sweetness	Bitterness
Group BC	4.31 ± 0.46^a^	4.81 ± 0.37^a^	4.5 ± 0.53^a^	4.5 ± 0.53^a^	4.63 ± 0.52^a^	4.56 ± 0.5^a^	1.81 ± 0.26^a^	4.13 ± 0.44^ab^	4.13 ± 0.23^b^	2.13 ± 0.64^b^
Group BB	4.44 ± 0.5^a^	4.75 ± 0.46^a^	4.5 ± 0.53^a^	4.38 ± 0.52^a^	4.38 ± 0.52^a^	4.31 ± 0.59^a^	1.69 ± 0.37^a^	4.56 ± 0.5^ab^	4.63 ± 0.44^a^	2.75 ± 0.46^a^
Group SC	4.56 ± 0.5^a^	4.75 ± 0.46^a^	4.44 ± 0.5^a^	4.5 ± 0.76^a^	4.5 ± 0.76^a^	4.38 ± 0.52^a^	1.75 ± 0.65^a^	4.06 ± 0.42^b^	4.25 ± 0.38^ab^	2.13 ± 0.58^b^
Group SB	4.44 ± 0.5^a^	4.88 ± 0.35^a^	4.31 ± 0.46^a^	4.63 ± 0.52^a^	4.63 ± 0.52^a^	4.5 ± 0.53^a^	1.94 ± 0.56^a^	4.63 ± 0.52^a^	4.44 ± 0.42^ab^	2.25 ± 0.53^ab^

Note

Means of score with different letters within each column were significantly different (*p* < .05) regarding cooking methods effect.

## 4 CONCLUSION

For fresh aquatic products such as crab, consumers pay more attention to quality comparison based on sensory evaluation. Thus, this study compared the taste quality of swimming crab cooked under four different conditions and combined with sensory evaluation. It aimed to provide a theoretical guidance for heat processing technology of swimming crab, so as to ensure the quality of final products and reduce energy consumption as much as possible. The results revealed that cooking methods affected edible crabs’ taste quality and sensory properties. As for heating medium factor, relatively higher contents of nonvolatile taste active components were obtained by steaming. The EUC value indicated that initial temperature of heating medium plays a decisive factor in the taste quality of cooked gonad. This might be, at least partly, related to extent of denaturation of protein. The mechanism was recommended for further study.

## CONFLICT OF INTEREST

The authors have declared that there is no conflict of interest to this work.

## ETHICAL APPROVAL

The experiment was performed in accordance with relevant institutional and national guidelines for the care and use of laboratory animals.
